# Unraveling the Engagement of Kinases to CRBN Through a Shared Structural Motif to Optimize PROTACs Efficacy

**DOI:** 10.3390/biom15020206

**Published:** 2025-02-01

**Authors:** Serena Rosignoli, Sara Giordani, Maddalena Pacelli, Giulia Guarguaglini, Alessandro Paiardini

**Affiliations:** 1 Centre for Regenerative Medicine “Stefano Ferrari”, Department of Life Sciences, University of Modena and Reggio Emilia, 41125 Modena, Italy; 2 Department of Biochemical Sciences “A. Rossi Fanelli”, Sapienza University of Rome, 00185 Rome, Italy; sara.giordani@uniroma1.it (S.G.); maddalena.pacelli@uniroma1.it (M.P.); 3 Institute of Molecular Biology and Pathology, National Research Council of Italy, c/o Department of Biology and Biotechnology, Sapienza University of Rome, 00185 Rome, Italy; giulia.guarguaglini@uniroma1.it

**Keywords:** PROTACs, kinases, E3 ligases, G-loop

## Abstract

PROteolysis TArgeting Chimeras (PROTACs) offer a therapeutic modality for protein target engagement, exploiting the ubiquitin–proteasome system to achieve precise degradation of a protein of interest. Recent advancements in understanding the structural biology of the CRL4A E3 ligase complex, particularly its recruitment of neo-substrates through the G-loop motif, have provided valuable insights into the optimization of PROTAC efficacy. This perspective delves into the molecular determinants governing PROTAC selectivity and degradation efficiency, with a specific focus on kinases showing distinct G-loop conformations. By employing computational approaches to predict ternary complexes, along with the identification of binding patterns, it is possible to address limitations posed by structural data scarcity, thereby enhancing rational design strategies.

## 1. Introduction

**Figure 1:**
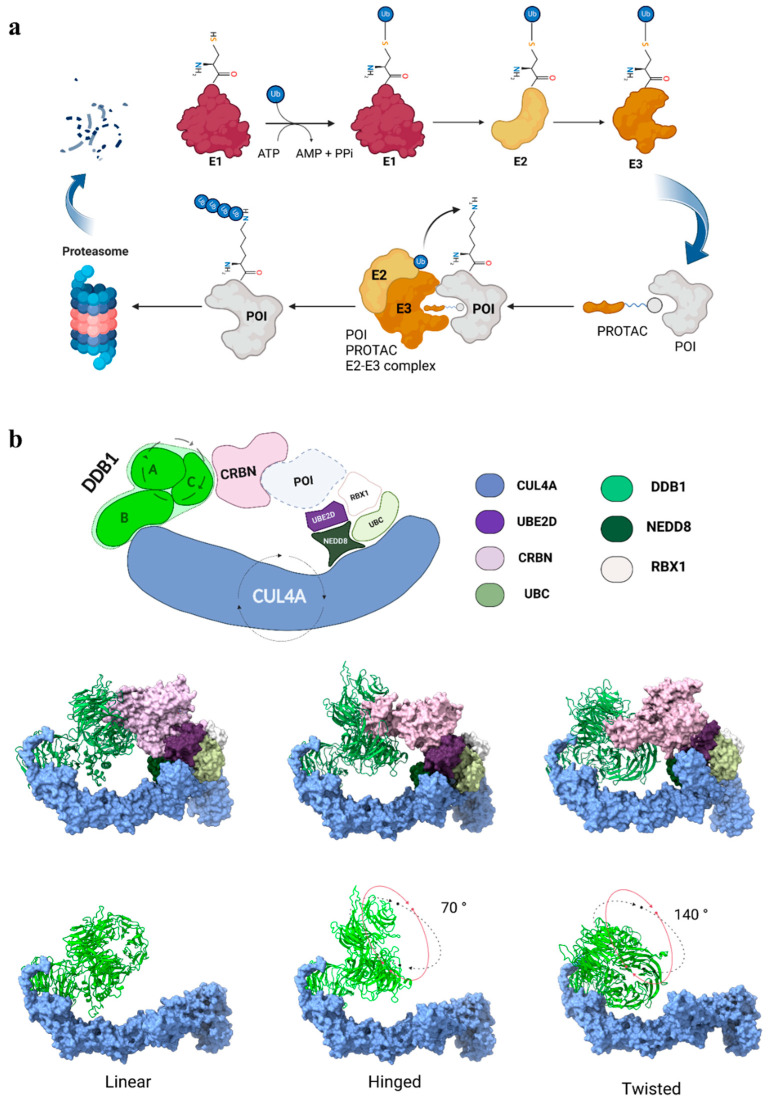
(**a**) Schematic representation of the ubiquitination process involving the ubiquitin-activating enzyme (E1), which activates ubiquitin in an ATP-dependent manner, through the formation of a thioester bond. The activated ubiquitin is transferred to the ubiquitin-conjugating enzyme (E2), which delivers ubiquitin to the ubiquitin ligase (E3) for substrate selection. E3 simultaneously binds the E2–ubiquitin complex and the substrate protein, facilitating the covalent attachment of ubiquitin to a lysine residue on the target protein, thereby marking it for proteasomal degradation. PROTACs (proteolysis-targeting chimeras) facilitate targeted degradation by serving as bifunctional molecules that recruit E3 ligases and bring them into proximity with the protein of interest (POI), promoting its ubiquitination and subsequent degradation. (**b**) Schematic and structural representation of the CRL4A^CRBN^ complex. DDB1 (A, B, C): DNA Damage-Binding Protein 1 subunits A, B, and C; CRBN: cereblon; POI: protein of interest; UBE2D: ubiquitin-conjugating enzyme E2; RBX1: RING protein; NEDD8: ubiquitin-like protein; UBC: polyubiquitin-C. Rotating arrows around DDB1 subunits A and C and around CUL4A represent the rotational movement of these subunits relative to subunit B and the opening motion involving CUL4A, respectively. In the rows below, the three stable conformations of the complex, i.e., linear, twisted, and hinged, are reported. In the linear orientation, used as a reference, the broad face of BPB aligns similarly to BPC, whilst the hinged and twisted are rotated ~70° and ~140° from the linear one, respectively. The entire complex (top row). DDB1-A-C subunits (bottom row) highlight the rotational differences between subunits A and C. Color legend: CRBN (light pink), CUL4A (light blue), UBE2D (purple), UBC (light yellow-green), RBX1 (white), and NEDD8 (dark green) are shown in the surface representation, while DDB1-A-B-C subunits (light green) are in the cartoon representation.

PROteolysis TArgeting Chimeras (PROTACs) are a promising therapeutic modality utilizing the ubiquitin–proteasome System (UPS) to selectively induce target protein degradation (TPD). PROTAC molecules consist of three scaffolds: a “warhead” that binds to the target protein, a second moiety that binds to the E3 ligase, and a linker connecting these two ligands. The purpose of PROTACs is to bring the target protein and the E3 ligase into close proximity, leading to the formation of a ternary complex. The latter facilitates the ubiquitination of the target protein by positioning it for efficient transfer of ubiquitin from the E2 enzyme, marking it for proteasomal degradation (Figure 1a) [1,2,3,4,5,6,7].

The modular design of PROTACs enables greater flexibility in drug development; by altering either the warhead or the E3 ligase recruiter, researchers can fine-tune the PROTACs’ activity, pharmacokinetics, and tissue selectivity, making them adaptable for various therapeutic applications. For this reason, PROTACs offer distinct advantages over classic drugs, including potentially higher selectivity and potency, as well as lower susceptibility to drug resistance [1]. Since PROTACs are bifunctional, they do not require a high degree of structural compatibility between the target protein and the E3 ligase, thus expanding the range of proteins that can be targeted, including those considered “undruggable”. Moreover, once a target protein is ubiquitinated and degraded, the PROTAC is released and recycled to degrade additional copies of the target, leading to potent and sustained protein degradation [6]. Finally, PROTACs can be engineered to recruit different E3 ligases (e.g., VHL, CRBN, or MDM2), allowing for tailored tissue-specific degradation and increased selectivity, which can help to overcome resistance mechanisms [7].

CRL4A, belonging to the “Really Interesting New Gene” (RING) E3 family, is a member of the largest known class of ubiquitin ligases, i.e., cullin-RING ubiquitin ligases (CRLs), and is one of the most leveraged for targeted protein degradation [8]. CRL4A is a protein complex composed of different units (Figure 1b). Cullin 4A (CUL4A) acts as the scaffolding core of CRL4A, as it recruits the UV-Damaged DNA Binding (DDB1) protein at the N-terminal domain (NTD) and the RING protein (Rbx1) at the C-terminal end (CTD) [9,10]. DDB1 acts as an adaptor protein for the recruitment of various substrate receptors (e.g., cereblon, CRBN), which are known as DDB1- and CUL4-associated factors (DCAFs) [11]. CRL4A is activated by neddylation, a covalent post-translational modification where the ubiquitin-like protein NEDD8 is covalently attached to CUL4A [12,13]. The induced conformational change makes Rbx1—the RING finger protein of CRL4A [14]—adept at recruiting the E2–ubiquitin-conjugating enzyme and positioning it correctly for the transfer of ubiquitin molecules to the substrate. The E2 enzyme’s active site, which carries the ubiquitin molecule, is positioned in close proximity to the substrate, facilitated by the scaffold function of CUL4A and the adaptor role of DDB1 [15]. DDB1 is composed of three WD-40 β-propellers (BPA, BPB, BPC), with BPA and BPC rotating with respect to BPB [16] and conferring flexibility to the protein. Notably, DDB1 allows for different orientations of its mobile BPB propeller, a feature essential for its function. Nevertheless, among the range of movements, some conformations are found to be the most stable, namely the “linear”, “hinged”, and “twisted” ones [17] (Figure 1b). The varied nature of DCAFs, combined with the mobility of DDB1, allows the CRL4A ligase complex to cover a substantial ubiquitination area, making it effective in degrading a wide range of target proteins.

**Figure 2:**
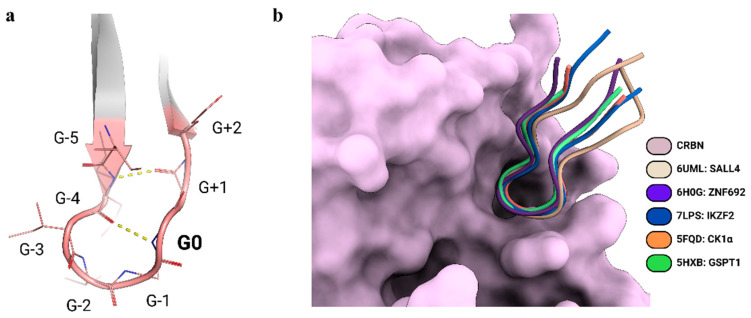
(**a**) Schematic representation of the G-loop (contacts are shown as dashed yellow lines), which consists of a β-hairpin loop with a glycine residue in a key position (G0), and (**b**) the superimposed G-loops from experimentally solved structures highlighting the interaction with CRBN.

Understanding the structure and degradation mechanism of the CRL4A complex has significantly advanced the rational design of PROTACs, for which medicinal chemistry exploration has been traditionally limited to the optimization of cell permeability and potency [18,19]. For example, in the case of the CRBN-binding drug thalidomide, as well as its derivatives pomalidomide and lenalidomide, a trial-and-error approach had been usually undertaken, where multiple linkers and attachment points were tested in conjunction with ligands for various E3 ligases [20,21,22]. Novel structural insights, coming from the analysis of the CRL4A complex with the target proteins, are enhancing design and structure-based optimization of PROTACs, the stability and half-life of the ternary complex, and degradation potency [23,24]. However, since determining the crystal structures of such complexes remains challenging [25], attention has increasingly shifted towards computational predictions, which may enable structure-based optimization in the absence of an experimentally determined structure [26]. Recent structural studies have revealed common features among the neo-substrates of CRBN when engaged with thalidomide-like degraders. A structural motif has been defined, known as the G-loop, which consists of a β-hairpin loop with a glycine in a key position (G0; Figure 2a) [27]. Around 1400 proteins in the human proteome feature a G-loop recognized by CRBN [28], some of which have also been structurally characterized (Table 1) (CK1α, PDB-ID: 5FQD [29]; GSPT1, PDB-ID: 5HXB [30]; ZNF692, PDB-ID: 6H0G [31]; SALL4, PDB-ID: 6UML [32]; IKZF2, PDB ID: 7LPS [33]) (Figure 2b). Although computational predictions may face some drawbacks, given the above-mentioned high dynamicity of the degradation machinery [34], this wealth of structural information on the CRL4A complex and, most importantly, the identification of shared patterns of interactions among its target proteins may be translated to the computationally-driven, rational design of PROTAC-mediated interactions.

With these advantages in mind, we aimed to identify the essential features enabling protein kinases with G-loops to be effectively targeted by CRBN-based PROTACs. Protein kinases are well-established targets in cancer therapeutics due to their central role in regulating cellular signaling pathways and their frequent dysregulation in cancer [35]. However, the clinical application of kinase inhibitors has faced significant challenges, including off-target toxicity [36], or the persistence of kinase-independent oncogenic functions that contribute to disease progression [37]. These limitations have prompted the development of alternative pharmacological strategies, such as covalent and allosteric inhibitors [38], which aim to enhance specificity and efficacy. Similarly, PROTACs offer a promising and alternative approach, potentially overcoming both enzymatic and non-canonical oncogenic effects [39].

## 2. Rationalizing Selectivity of CDK Kinases-Targeting PROTACs

A growing body of research has shown that even closely related kinases can exhibit different degradation efficiencies when targeted by the same PROTAC [40]. For instance, cell cycle-associated kinases such as CDK1, CDK2, CDK4, and CDK6 show degradation patterns influenced by the cell cycle stage [41], although these degradation efficiencies vary across structurally similar kinases, as observed with PROTAC TL12-186 [42].

Computational models by Bai et al. (2022) [26] propose that the degradation effectiveness depends heavily on the ubiquitination efficiency of each target, which can vary even among homologous kinases, due to differences in the availability or accessibility of ubiquitination sites. Target-specific degradation may hinge on whether particular lysine residues within a target are “totally”, “partially”, or “not responsible” for ubiquitination, as demonstrated by site-directed mutagenesis of CDK5, CDK2, and CDK9. Therefore, the success of PROTACs is influenced by several factors, including the cooperativity of ternary complex formation, which varies based on linker length, type, and the nature of the protein–protein interactions (PPIs) involved. While cooperativity has been noted in most cases, both non-cooperative and anti-cooperative interactions have been reported [43], suggesting complexity in ternary complex stability and selectivity.

In addition to the primary van der Waals and hydrogen bonding interactions with the G-loop, CRBN can engage in additional PPIs with other domains of the neo-substrate that extend beyond the G-loop. Understanding these interactions could provide a rational basis for designing selective PROTACs capable of leveraging CRBN’s adaptability to target a wider array of substrates effectively. Modulating the linker length significantly influences the spatial positioning and flexibility of PROTACs. For instance, CRBN-based PROTACs targeting Aurora-A show variable efficacy depending on linker length [44]. An optimal linker ensures precise alignment of the components, enhancing interactions and promoting efficient target protein degradation, while excessively short or long linkers disrupt this alignment, reducing efficacy.

A paradigmatic example is found for CDK4 and CDK6, for which previous studies have demonstrated that the latter, despite the high similarity of the two kinases, is more efficiently degraded by the same PROTAC molecules [45]. Given that the warhead ligand, i.e., palbociclib [46], is a known ATP-competitive inhibitor of both kinases with comparable affinity, and the compound affinity is not affected by linker addition, the differences in degradation efficiency may be attributed to neo-interactions with CRBN, as well as to the PROTAC’s linker length. Among the five PROTACs developed to target CDK4 and CDK6, by conjugating pomalidomide and palbociclib with varying linker lengths, the one with the longest linker (261 g/mol; PROTAC-6) showed the highest effectiveness, but also a marked selectivity for CDK6. Therefore, structural models in this case may be pivotal to uncovering the rationale underlying these observed differences whilst providing insights that could be applied to the design of more precise and effective kinase-targeting PROTACs.

**Figure 3:**
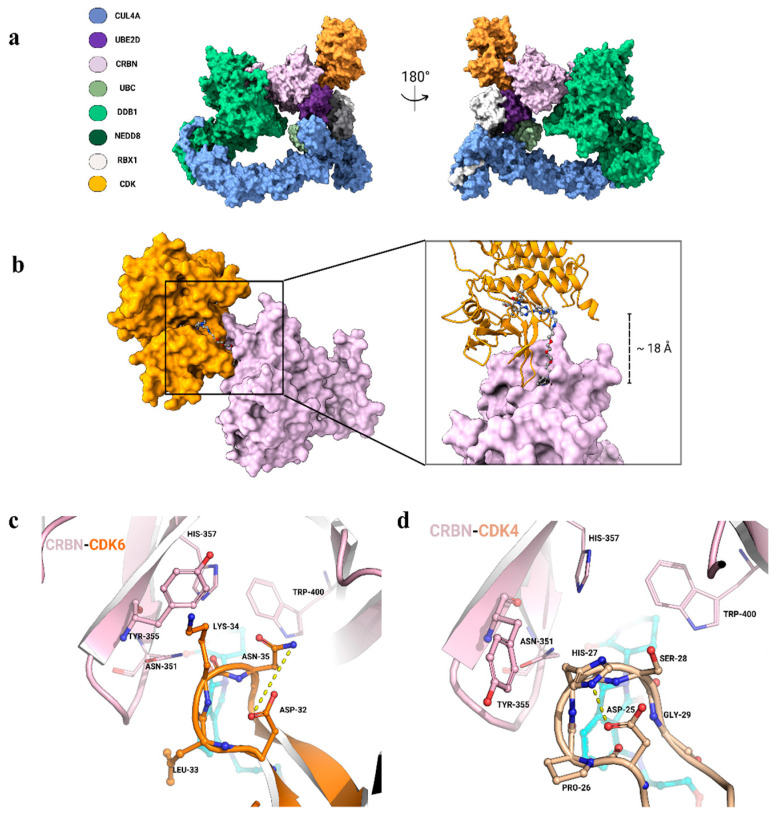
(**a**) Models of the CDK kinases’ interaction with the CRL4A^CRBN^ complex in the hinged conformation; “CDK” label represents both CDK4 and CDK6. (**b**) Model of the productive ternary complex when PROTAC-6 is docked into the cleft (coloring scheme is consistent with panel). (**c**) Comparison of the PROTAC-mediated binding mode (contacts shown in dashed yellow lines) between (**c**) the CDK6 G-loop (32-DLKNG-36, orange) and (**d**) the CDK4 G-loop (25-DPHSG-29, beige), which are predicted to interact with CRBN (light pink) in a cleft formed by residues Asn351, Tyr355, His357, and Trp400.

In an attempt to rationalize such observations, we recapitulated the dynamics of the CRL4A complex by combining the recently solved complexes of CRL4A complex (PDB-ID: 8B3I [47]) with the complexes of DDB1:CRBN in the three conformations: linear, twisted, and hinged (PDB-IDs [17] 8D7U, 8D7V, 8D7W, respectively). Modeling was performed through the integrated use of AlphaFold3 predictions [48,49], combined with protein–protein interface refinement strategies [50] and a tethered docking protocol [51]. CDK4 and CDK6, as in the case of Ck1α, likely interact with the CRL4A complex, adopting a “hinged” conformation, and with their G-loops (CDK6: “32-DLKNG-36” and CDK4: “25-DPHSG-29”) fitting in the same pocket (Figure 3a). In this orientation, the final model reveals that the binding site of CRBN and the kinase active site are approximately 18 Å apart. This spatial arrangement positions both ligands near the exit of their respective protein-binding sites, forming a “tunneling” cleft that can be occupied by the PROTAC linker (Figure 3b). Consistent with the observed correlation between linker length and efficacy, only PROTAC-6 appears to be optimal for the formation of a productive ternary complex. The obtained model supports the PROTAC-mediated binding of both CDK6 and CDK4, with their G-loops fitting into the cleft between the phthalimide ring and CRBN, consistent with known interactions involving CRBN residues Asn351, His357, and Trp400 (Figure 3c,d). However, the G-loop of CDK6 appears more adaptable to the CRBN pocket and forms additional contacts with other CRBN residues. Specifically, Leu33 of CDK6 may help in creating a hydrophobic environment around the phthalimide ring, whilst Lys34 in CDK6 may orient towards contacting Tyr355 of CRBN through a π-cation interaction. This favorable interaction is also promoted by CDK6 Asn35, which contributes by freeing Lys34 hydrogen bonding with Asp32 (Figure 3c). The same configuration is not observed in the CDK4 loop, in which His27, packed back on the loop and stabilized by the interaction with Asp25, cannot be engaged in contact with Tyr355 of CRBN. This observation, along with the presence of Proline in the CDK4 loop, suggests that the latter is less dynamic and unable to properly accommodate the CRBN binding cleft (Figure 3c). Consistently, none of the known CRBN interactors contain a proline residue in their G-loops, indicating that loop flexibility may play a key role in binding. Finally, the G-loop of CDK6 more closely resembles that of CK1α, especially for the presence of asparagine (Figure 3d) and of an hydrophobic residue at the G-1 and G-3 positions, respectively. These structural differences likely underlie the higher selectivity observed in PROTAC efficiency toward CDK6 over CDK4.

## 3. Conclusions

PROTACs have emerged as powerful tools for targeted protein degradation, with the potential to address undruggable targets through the ubiquitin–proteasome system. By integrating structural insights and computational modeling, this perspective highlights how specific design elements—such as linker length and neo-interactions—can be leveraged to enhance the selectivity and efficacy of CRBN-based PROTACs. In targeting kinases, the structural characterization extends to identifying a conserved binding motif within the N-lobe, a structural feature whose specific amino acid composition has been shown to play a critical role in CRBN binding. Understanding the variability and functional implications of this G-loop composition across different kinases will enable tailored targeting strategies. Comprehensively, this approach can shed light on the mechanisms of CRBN recruitment, both in physiological contexts and when mediated by thalidomide-like compounds.

Future work could explore the broader applicability of these findings to other undruggable targets and investigate novel strategies for modulating CRBN recruitment using thalidomide-like compounds. Furthermore, integrating these design principles into high-throughput screening and machine learning frameworks may accelerate the discovery of next-generation PROTACs. Some prospective directions may be: (i) Map G-loop conformational diversity across the kinome using structural databases and computational models; and (ii) assessing how G-loop sequences and dynamics impact PROTAC selectivity and degradation kinetics.

## Figures and Tables

**Table 1:** The proteins featuring a G-loop binding mode, whose structures have been experimentally characterized.

Targeted Protein	PDB ^1^	Compound	Ref.
Casein kinase 1 (CK1α)	5FQD	Lenalidomide	[29]
GTP-binding subunit ERF3A (GSPT1)	5HXB	CC-885	[30]
Zinc finger protein 692 (ZNF692)	6H0G	Pomalidomide	[31]
Sal-like protein 4 (SALL4)	6UML	Pomalidomide	[32]
Zinc finger protein Helios (IKZF2)	7LPS	ALV1	[33]

^1^ The resolution method for all structures was X-ray diffraction.

## Data Availability

The 3D structures of the models are available upon request.
